# Large Fibrous Connective Tissue Reduces Oxidative Stress to Form a Living Cell Scaffold in Adipose Grafts

**DOI:** 10.3390/antiox14030270

**Published:** 2025-02-26

**Authors:** Qiang Yue, Zilong Cao, Tiran Zhang, Ningbei Yin, Liqiang Liu

**Affiliations:** Plastic Surgery Hospital, Chinese Academy of Medical Sciences and Peking Union Medical College, Beijing 100144, China; yueqiang@student.pumc.edu.cn (Q.Y.); caozilong@pumc.edu.cn (Z.C.); tiranzhang@psh.pumc.edu.com (T.Z.)

**Keywords:** fat grafting, mitochondria, reactive oxygen species, hypoxic, fibrous connective tissue

## Abstract

This study aimed to investigate the mechanisms by which large fibrous connective (LFC) tissue enhances fat graft survival in fat transplantation. A block fat graft model demonstrated that intact fat containing LFC showed significantly higher survival rates compared with liposuctioned fat. In the center of intact grafts, viable fat cells surrounded the LFC, forming a mesh-like living tissue structure. Proteomics of the extracellular matrix (ECM) adjacent to LFC (ALFC) and distant to LFC (DLFC) revealed significant differences in mitochondrial aspects. Staining of LFC tissue showed that it contains a large number of blood vessels and mitochondria, and exhibits stronger antioxidant capacity (*p* < 0.05) compared with adipose tissue. By mixing LFC with liposuctioned fat and transplanting into nude mice, histological sections showed that LFC promotes SOD1 expression, enhances respiratory chain RNA expression, and reduces ROS and inflammation. Pure mitochondrial-assisted fat transplantation only reduced short-term graft inflammation without improving long-term survival rates. In conclusion, LFC enhances long-term survival rates by reducing oxidative stress in fat grafts and forming a center for fat cell survival, thereby overcoming distance limitations. This represents a novel mechanism distinct from classical fat survival models and provides a reference for clinical practice.

## 1. Introduction

Fat grafting is widely applied in soft tissue reconstruction and aesthetic surgery [1]. Beyond the commonly used technique of particle fat grafting with Coleman fat [1,2], block fat transplantation is an alternative approach, especially suitable for localized concavities resulting from trauma or tumor excisions, where targeted filling is preferred over diffuse injections [2]. Despite various modifications, a significant limitation of fat grafting remains its complications, which include calcification, nodule, and reduced volume retention rate. These complications are primarily due to post-graft ischemia and hypoxia [3].

The survival of fat grafts is traditionally explained by Yoshimura’s three-zone theory [4]. Consequently, fat grafts larger than 1.5 mm in radius tend to experience necrosis, reducing their volume retention, which typically ranges from 30 to 80% [5]. This leads to all fat grafts facing the same fate: central cells inevitably die, while peripheral cells partially survive, forming a shell-like survival pattern. In fact, as early as 1955, Lyndon A. Peer discovered that approximately 45% of the volume of grafted particulate fat would be absorbed [6]. Subsequently, researchers have continuously attempted to improve survival rates, including via the use of low-pressure liposuction, low-speed centrifugation, and by adding adipose-derived stem cells during transplantation; however, none of these methods have significantly improved the central necrosis of the grafts [7]. Fascia-fat flap transplantation has shown high survival rates since its introduction [3], but the harvesting process is more invasive, so it is primarily used for procedures requiring specific fat shapes, such as facial contouring. Due to ethical constraints, it is challenging to re-biopsy patients after intact fat grafting, which has led to a lack of reported mechanisms explaining the high survival rates of these grafts.

Our clinical experience indicates that free block fat without dermis can achieve satisfactory volume retention [2]. To further investigate the survival mechanisms of transplanted fat, we studied discarded adipose tissue from abdominal wall plastic surgeries. Surprisingly, block fat not only exhibited higher volume retention rates compared with particle fat but also showed a mesh-like survival pattern—viable adipocytes surrounding large fibrous connective tissue (LFC). This pattern deviates from Yoshimura’s three-zone theory and known fat grafting models. LFC, a component of the extracellular matrix (ECM) of fatty tissue, contains elastic fibers, collagen, vasculature, nerves, and a range of secreted proteins and adipokines [8,9,10]. While LFC is often considered detrimental in particle fat grafting and is removed [11,12], it is preserved in block fat grafting. Given the observed pattern of adipocyte survival around LFC, we hypothesized that LFC might have a beneficial role in fat grafting.

In this study, we initially evaluated the high survival rate of intact fat grafts based on their histological features. We then investigated the reasons for this high survival rate by examining adipose-derived stem cells and the extracellular matrix. Next, we analyzed differences between fat with LFC and fat without LFC to explore how redox status, inflammation, and energy parameters contribute to enhanced fat graft survival through LFC. Finally, we validated our hypothesis using mitochondrial transplantation and LFC-assisted fat grafting. Our results reveal a promising method to improve fat graft survival by incorporating LFC.

## 2. Materials and Methods

### 2.1. Graft Preparation

The collection and use of adipose tissue were approved by the Ethics Committee of the Plastic Surgery Hospital of the Chinese Academy of Medical Sciences (No. ZX201843). All fat tissue used in this study was obtained from discarded fat collected during surgical procedures. Written informed consent was obtained from all participants prior to their inclusion in the study. The methods employed in this study were conducted in strict accordance with the principles outlined in the Helsinki Declaration.

#### 2.1.1. Particle Fat and Block Fat

Abdominal adipose tissue was collected from abdominoplasty procedures in healthy female patients (BMI: 18–24 kg/m^2^, age: 25–45 years). Using Scarpa’s fascia as a boundary, deep fat was separated and discarded. For particle fat (PF), superficial fat was aspirated using a low-vacuum suction device consisting of a 50 mL syringe and a 2.5 mm liposuction cannula, and LFC was manually removed. For block fat (BF), superficial fat tissue was trimmed into 0.3 mL spherical pieces and washed twice with PBS.

#### 2.1.2. LFC-Included Group

Adipose tissue from healthy female patients (BMI: 18–24 kg/m^2^, age: 25–45 years) undergoing liposuction was collected. We gently stirred the fat suspension using a Pasteur pipette (Beyotime, Shanghai, China), and the tissue wrapped around the basilar canal was collected as adipose tissue adjacent to LFC (ALFC). The ALFC was minced by scissors and mixed back with the lipoaspirate, which was defined as the LFC-included group. The fat suspension without LFC was defined as adipose tissue distant to LFC (DLFC) which served as the control group.

The adipose tissue obtained in Section 2.1.1 and Section 2.1.2 was washed 2–3 times with PBS and centrifuged at 1000× *g* (3 min) using a centrifuge (Thermo Scientific, Waltham, MA, USA) to remove liquids and oils before use.

#### 2.1.3. ECM Extraction

The washed fat was homogenized at 25,000 rpm for 2–3 min and centrifuged at 3500× *g* for 3 min. The supernatant was discarded, and the remaining material was decellularized as described previously [13]. Briefly, the material was sequentially soaked and shaken in 0.5 M NaCl, 1 M NaCl, sterile distilled water, and 1% Triton X-100 solution (Sigma, Saint Louis, MO, USA) (37 °C, 100 rpm) for 4 h, 4 h, 10 h, and 48 h, respectively. It was then rinsed three times with sterile distilled water (30 min for each rinse), shaken in 99% isopropanol for 6 h, and washed again with sterile distilled water. Sterilization was performed with 75% ethanol (three cycles), and finally, the decellularized adipose matrix was lyophilized at −80 °C for 72 h.

### 2.2. Mitochondrial Transplantation

#### 2.2.1. Mitochondrial Extraction

Mitochondria were extracted from adipose-derived stem cells (ADSCs) (Passage 3) using a mitochondrial extraction kit (Abcam, Cambridge, UK). ADSCs (5 × 10^7^) were collected and homogenized on ice. The cell homogenate was centrifuged at 4 °C, 1000× *g* for 10 min twice. The supernatant was collected and then centrifuged at 4 °C at 12,000× *g* for 15 min. The resulting mitochondria were retained and resuspended in 100 μL of PBS.

#### 2.2.2. Mitochondrial Concentration Detection

Mitochondrial suspension (2 μL) was mixed with the BCA working solution (Sigma, USA) and incubated at 37 °C for 30 min. The absorbance at 562 nm was measured using a microplate reader (PerkinElmer, Hopkinton, MA, USA), and the concentration was expressed in μg/μL.

#### 2.2.3. Mitochondrial Labeling

DLFC obtained in Section 2.1.2 was mixed with MitoBright Green LT (Dojindo, Kyushu, Japan) at a volume ratio of 1:5 and incubated at 37 °C for 1 h. Freshly extracted mitochondria were mixed with MitoBright Red LT (Dojindo, Japan) diluted 1000 fold in high-glucose Dulbecco’s modified Eagle’s medium (DMM) at a volume ratio of 1:10 and incubated for 30 min. The fluorescent labeling was observed using a confocal microscope (Leica, Weztlar, Germany).

Fluorescently labeled particle fat (4 mL) was mixed with 400 μg (400 μL) and 200 μL (200 μL) extracted mitochondria, to form higher and lower concentration mitochondrial transplantation groups (MT group). The mixtures were incubated at 37 °C for 30 min. The control group (CT group) consisted of 4 mL of DLFC mixed with 400 μL and 200 μL of PBS. The entry of free mitochondria into cells was observed using a confocal microscope.

### 2.3. Animal Experiments

All animal experiments were carried out by the National Institutes of Health guide for the care and use of laboratory animals (NIH Publications No. 8023, revised 1978), and were approved by The Chinese Academy of Medical Sciences and Peking Union Medical College Animal Ethics Committee (Approval No. 2023(2); approval date: 3 April 2018). Eighty-seven female Balb/c nude mice (6 to 8 weeks old, 10,125) were purchased from Beijing Vital River Laboratory Animal Technology Company. All mice were anesthetized with gas maintenance anesthesia before transplantation. All mice were humanely euthanized by carbon dioxide inhalation prior to tissue collection, in accordance with approved guidelines. To control for extraneous variables, the grafts (including fat and ECM) transplanted into the same nude mouse are sourced from a single individual, while grafts for different nude mice may be sourced from different individuals. Additionally, during the graft injection, the force/speed on both sides is maintained consistently.

#### 2.3.1. PF and BF Grafting

Eighteen mice were used, and each side received one sample. A 1 cm incision was made on the back using ophthalmic scissors. Subcutaneous tissue was dissected on the right side, and 0.3 mL of block fat was implanted. On the left side, 0.3 mL of particle fat was injected subcutaneously using an 18-gauge needle. Animals were euthanized at 3 weeks, 6 weeks, and 12 weeks (n = 6 at each time point).

#### 2.3.2. ALFC-ECM and DLFC-ECM Grafting

Fifteen mice were used, and each side received two samples (cephalic: DLFC-ECM, caudal: ALFC-ECM). At each injection site, 5 mg (dry weight) of lyophilized ECM was soaked in 200 μL of normal saline and thoroughly cut. The suspension was injected subcutaneously into the dorsal region using 18-gauge needles. Animals were euthanized at 2 weeks, 4 weeks, and 8 weeks (n = 5 at each time point).

#### 2.3.3. Mitochondrial Transferred Fat Grafting

For higher concentration transplantation, twenty-seven mice were used, and each side received two samples. Using an 18-gauge needle, 0.3 mL of fat was injected into the subcutaneous region of the back (left: CT group, right: MT group). Eighteen mice were euthanized at 1 weeks, 4 weeks, 6 weeks, and 12 weeks (n = 6 at each time point), and three mice were euthanized at 2 days. For lower concentration transplantation, nine mice were euthanized at 1 weeks, 6 weeks, and 12 weeks (n = 3 at each time point).

#### 2.3.4. LFC-Included Fat Grafting

Eighteen mice were used, and each side received one sample. Using an 18-gauge needle, 0.3 mL of fat was injected subcutaneously into the back (left: LFC-included group, right: control group). Samples were taken at 1 week, 6 weeks, and 12 weeks (n = 6 at each time point).

### 2.4. Histology

The collected adipose tissue was washed with PBS to remove blood, blotted dry to remove excess moisture, and subsequently fixed in 10% neutral buffered formalin solution (Solarbio, Beijing, China) for 48 h. Then, the tissue was dehydrated in 95% ethanol for 24 h. Routine processing included clearing, paraffin infiltration, and embedding. The adipose tissue was then sectioned into 5-micron-thick slices. These sections were stained with hematoxylin and eosin (HE staining). Histological parameters were independently evaluated by two pathologists and included structural integrity (nucleated adipocytes with intact cell membranes), necrotic areas, cysts and vacuoles, inflammation, and fibrosis. Histological scoring was based on the percentage of each component in the sample area: 0 (absent, <5% area), 1 (minimal, 5 to 25% area), 2 (minimal to moderate, 26 to 50% area), 3 (moderate, 51 to 75% area), 4 (moderate to extensive, 76 to 95% area), or 5 (extensive, >95% area) [13].

The sections were deparaffinized in xylene, dehydrated through a graded series of ethanol, subjected to antigen retrieval by boiling, and blocked for non-specific binding sites. The following primary antibodies were then incubated overnight at 4 °C: anti-perilipin-1 (ab3529, Abcam), CD31 (ab28364, Abcam), anti-HSP60 (ab190828, Abcam), anti-Ly6G (ab307167, Abcam), and anti-superoxide dismutase 1 (SOD1) (ab308181, Abcam). Appropriate secondary antibodies were applied for immunohistochemistry (IHC) and immunofluorescence (IF) detection. Images of the sections were captured using a microscope. The ImageJ software (ImageJ/Fiji, https://imagej.net/ij/, NIH, Bethesda, MD, USA) was used to analyze the percentage area of positive regions and the mean fluorescence intensity. To ensure the specificity and reliability of the IF/IHC results, we used a secondary antibody-only control. Any non-specific signals in the control group were observed and analyzed under the microscope (Leica, Weztlar, Germany).

### 2.5. Electron Microscopy

#### 2.5.1. Scanning Electron Microscopy (SEM)

The lyophilized ECM was fixed with 2.5% glutaraldehyde. Graded dehydration was performed using the following series of ethanol solutions: 20%, 50%, 70%, 90%, and 100% ethanol, 10 min each, 100% twice. The samples were then lyophilized and sputter-coated with gold for 300 s. The pore diameter (μm) was measured using Image-Pro Plus 6.0 software (Media Cybernetics, Inc., Rockville, MD, USA) with a 100 μm (×300) scale as the reference.

#### 2.5.2. Transmission Electron Microscopy (TEM)

Fresh fat specimens (1 mm^3^) were immediately immersed in a 4 °C fixative containing 2.5% glutaraldehyde for 2–4 h and 1% osmium tetroxide for 2 h, followed by a series of ethanol gradient dehydration steps. The tissue was infiltrated with epoxy resin, gradually replacing the ethanol, and ultimately polymerized at 60 °C. Ultrathin sections (50 nm) were prepared and stained with uranyl acetate and lead citrate. A transmission electron microscope (TEM-1400plus, JEOL, Tokyo, Japan) was used to observe.

### 2.6. Mitochondrial Membrane Potential (MMP) Detection

To intuitively demonstrate the relationship between MMP and tissue structure, the MMP was detected using the JC-1 assay kit (Merck, Darmstadt, Germany) according to the manufacturer’s instructions. Freshly extracted mitochondria were incubated with JC-1 working solution at 37 °C for 30 min, collected by centrifugation at 4 °C (12,000× *g*, 5 min), and resuspended in high-glucose DMEM for measurement. Adipose tissue was cut into 0.5 mm diameter particles, mixed with JC-1 reagent, and incubated at 37 °C for 30 min. After being washed with JC-1 wash buffer, the adipose and extracted mitochondria were visualized under a fluorescence microscope (Leica, Weztlar, Germany). The ratio of red (JC-1 aggregate) fluorescence to the sum of green (JC-1 monomer) and red fluorescence was used to evaluate the MMP.

### 2.7. Measurement of Oxidative Stress and Antioxidants in the Graft

After fat transplantation, the grafts were harvested at different weeks. Fresh tissue supernatant homogenate was prepared, the DHE probe (Solarbio, China) add and the absorbance of the samples measured using a microplate reader (PerkinElmer, Waltham, MA, USA). Simultaneously, the protein concentration of the supernatant was quantified using the BCA method (Beyotime, China). The ratio of absorbance value to protein concentration (mg/protein) represents the relative intensity of reactive oxygen species (ROS).

The adipose antioxidant indexes for total antioxidant capacity (T-AOC), malondialdehyde (MDA) and glutathione (GSH) were determined using commercial test kits (Solarbio, Beijing, China), according to the instructions from the manufacturer (Thermo Scientific, Waltham, MA, USA).

### 2.8. Measurement of Adenosine Triphosphate (ATP)

ATP in the grafts (n = 6) was measured using an ATP detection kit. Samples (100 mg) were transferred to a grinding tube added with 1 mL of ice-cold grinding solution. The sample absorbance was measured according to the kit instructions (Solarbio, China) using a spectrophotometer (Thermo Scientific, Waltham, MA, USA).

### 2.9. Oxygen Level Measurement in Grafts

The oxygen concentration (μmol/L) was measured using a microelectrode (Unisense, Denmark). To ensure accuracy due to potential electrode drift, calibration is performed before each measurement. The calibration procedure involves the following: (1) Standard curve preparation: Measure the electrode voltage in a deoxygenated water sample (prepared by dissolving 0.8 g of sodium hydroxide and 1.76 g of sodium ascorbate (Sigma, USA) in 100 mL of double-distilled water). Then, measure the voltage again in oxygen-saturated water with oxygen at the same temperature as the sample being tested (achieved by bubbling air through 100 mL of double-distilled water at 37 °C for 3 min). Use these voltages to establish a standard curve for oxygen concentration under deoxygenated and saturated conditions at the specific temperature. (2) Sample measurement: Measure the voltage in the sample solution with the calibrated electrode and convert this voltage to oxygen concentration using the established standard curve. Under full gas anesthesia, a 1 cm incision was made near the graft. A Clark oxygen electrode with a tip diameter of 100 μm was inserted into the graft to a depth of approximately 0.5 cm. Once the electrode reading stabilized, the oxygen level was recorded (n = 6).

### 2.10. qRT-PCR Analysis

Total RNA was extracted by mirVana^TM^ RNA Isolation Kit (AM1561). The concentration and OD 260/OD 280 were determined by NanoDrop 2000 spectrophotometer (Thermo Scientific, Waltham, MA, USA). The tested RNA was reverse-transcribed into cDNA. The PerfectStartTM Green qPCR SuperMix kit was used for the reaction on the LightCycler^®^ 480II fluorescence quantitative PCR instrument (Roche, Basel, Switzerland). The gene expression levels were calculated using the 2^−ΔΔCT^ method. The primer sequences are shown in Supplemental Materials Table S1.

### 2.11. In Vitro Experiments

Subcutaneous fat tissue from abdominoplasty specimens was processed. Specifically, 1 mm radius fat tissue adjacent to the LFC was excised as ALFC-fat, and 1 mm radius fat tissue from the center of the adipocyte lobules distant from the LFC was excised as DLFC-fat. The ALFC-fat and DLFC-fat were digested with 0.075% type I collagenase (Sigma, USA) at 37 °C for 30 min to obtain ALFC-ADSCs and DLFC-ADSCs, respectively.

#### 2.11.1. Hypoxic Treatment of Adipose Tissue

ALFC-fat and DLFC-fat were placed in 10 cm diameter culture dishes and submerged in 20 mL of low-glucose DMEM without serum (Hyclone, Logan, UT, USA). The dishes were then placed in a 1% oxygen volume hypoxic incubator (Esico, Singapore) maintained at 37 °C, 5% CO_2_, and saturated humidity. Samples were taken at 3 and 7 days, fixed, and sectioned (n = 5). Perilipin immunofluorescence staining was performed, and the percentage of viable cell area was quantified in 3 high-power fields per slice.

#### 2.11.2. Dedifferentiation of Adipocytes

The dedifferentiation of ALFC-fat and DLFC-fat was conducted following previously reported procedures [13], which generally involved inducing the dedifferentiation of adipocytes under hypoxic conditions and observing the morphological change (adipocyte intracellular lipid droplet morphology and content, cell size, cell shape, and adhesion status) and the proportion of dedifferentiated cells (n = 5).

#### 2.11.3. Evaluation of ADSCs Activity with CCK-8

The activity of ADSCs was tested by CCK-8 at the same time every 2 days (n = 5). CCK8 working solution (100 μL, CCK8:DMEM = 1:9) was added and incubated at 37 °C for 2 h. The OD value at a wavelength of 450 nm was measured by ELISA (Thermo Fisher Scientific, Waltham, MA, USA).

#### 2.11.4. ADSCs Migration

ALFC-ADSCs and DLFC-ADSCs (1 × 10^5^ cells/well) were seeded onto the upper chamber of the transwell (Corning, 3422). DMEM with and without 20% FBS was added with a volume of 200 μL and 500 μL to the upper and lower chambers, respectively. The plates were incubated at 37 °C in 5% CO_2_ for 24 h. Cells were fixed with 4% paraformaldehyde and stained with crystal violet for 20 min. Cells that did not migrate through were gently removed using a cotton swab. Migrated ADSCs were analyzed using a light microscope (n = 5).

#### 2.11.5. Tri-Lineage Differentiation

##### Adipogenic Induction

ADSCs (passage 3, 2 × 10^4^ cells per well) were seeded in a 6-well plate and cultured until they reached 100% confluence. Adipogenic induction was performed using the adipogenic differentiation kit (HUXMD-90031, Oricell, Roseland, NJ, USA) by alternating between induction solution A for 3 days and solution B for 1 day. The normoxic group was induced under 21% oxygen, while the hypoxic group was induced under 1% oxygen. After 21 days, lipid droplet formation was observed after fixing the cells with 4% paraformaldehyde (Biosharp, China) for 20 min. Oil Red O staining was performed and lipid droplet formation was observed and photographed under a microscope. The adipogenic area ratio was calculated using Image J (ImageJ/Fiji, NIH, Bethesda, MD, USA) (n = 5).

##### Osteogenic Induction

ADSCs (passage 3, 2 × 10^4^ cells per well) were seeded in a 6-well plate and cultured in the low-glucose complete medium under standard conditions until they reached 70% confluence. Osteogenic induction was performed using the osteogenic differentiation kit (HUXXC-90021, Oricell, Santa Clara, CA, USA) for 21 consecutive days. After induction, cells were fixed with 4% paraformaldehyde and stained for alkaline phosphatase activity (C3250S, Beyotime, Shanghai, China) (n = 5).

##### Chondrogenic Induction

ADSCs (4 × 10^5^ cells) were placed in a 15 mL centrifuge tube and induced to differentiate into chondrocytes using the chondrogenic differentiation kit (HUXMD-90041, Oricell, USA) for 21 consecutive days. Once chondrogenic pellets with a diameter of 1.5–2 mm formed, they were fixed and processed for histological sectioning. Alcian blue staining was performed to assess chondrogenesis (n = 5).

### 2.12. Transcriptome Sequencing

The total RNA of the sample (n = 3) from mice was extracted using the TRIzol reagent (Invitrogen, Carlsbad, CA, USA). RNA purity and quantification were evaluated using the NanoDrop 2000 spectrophotometer (Thermo Scientific, Waltham, MA, USA). RNA integrity was assessed using the Agilent 2100 Bioanalyzer (Agilent Technologies, Santa Clara, CA, USA). The transcriptome sequencing was conducted by OE Biotech Co., Ltd. (Shanghai, China). PCA analysis was performed using R (v 3.2.0) to evaluate the biological duplication of samples. Differential expression analysis was performed using the DESeq2 [14]. Q value <0.05 and foldchange (FC) value ≥1.5 or ≤1/1.5 were set as the threshold for significantly different expressed genes (DEGs). Based on the hypergeometric distribution, GO [15] and KEGG [16] pathway enrichment analysis of DEGs were performed to screen the significantly enriched term using R (v 3.2.0). Gene set enrichment analysis (GSEA) was performed using GSEA software (https://www.gsea-msigdb.org/gsea/login.jsp, accessed on 21 February 2025).

### 2.13. Proteomic Identification

The ECM extraction method was performed using the enzyme-free technique as previously reported. The total protein in ECM was extracted and divided for total protein concentration measurement and trypsin enzymolysis. After desalting the enzymolysis peptide segment, the samples were identified by LC-MS/MS (4D-DIA). Data were imported to Spectronaut Pulsar 18.4 (Biognosys, Zurich, Switzerland) for analysis. The difference screening conditions were *p* value <0.05 and FC value ≥1.5 or ≤1/1.5.

### 2.14. Statistical Analysis

To ensure that the statistical software complies with standards, all data were analyzed using SPSS (SPSS Statistics software 27.0.x; IBM, New York, NY, USA) and expressed as the mean ± standard deviation (SD). The test of normality was performed before the analysis of the unpaired Student’s *t*-test. Student’s *t*-test was used to determine the significance of differences between two groups. When conducting multiple *t*-tests on the same dataset, the Bonferroni correction was employed post hoc to control for Type I error. Statistically significant differences were indicated as follows: *, *p* < 0.05; **, *p* < 0.01; and ***, *p* < 0.001.

## 3. Results

### 3.1. Survival of Adipocytes Around the LFC in Block Fat Grafting

**Figure 1:**
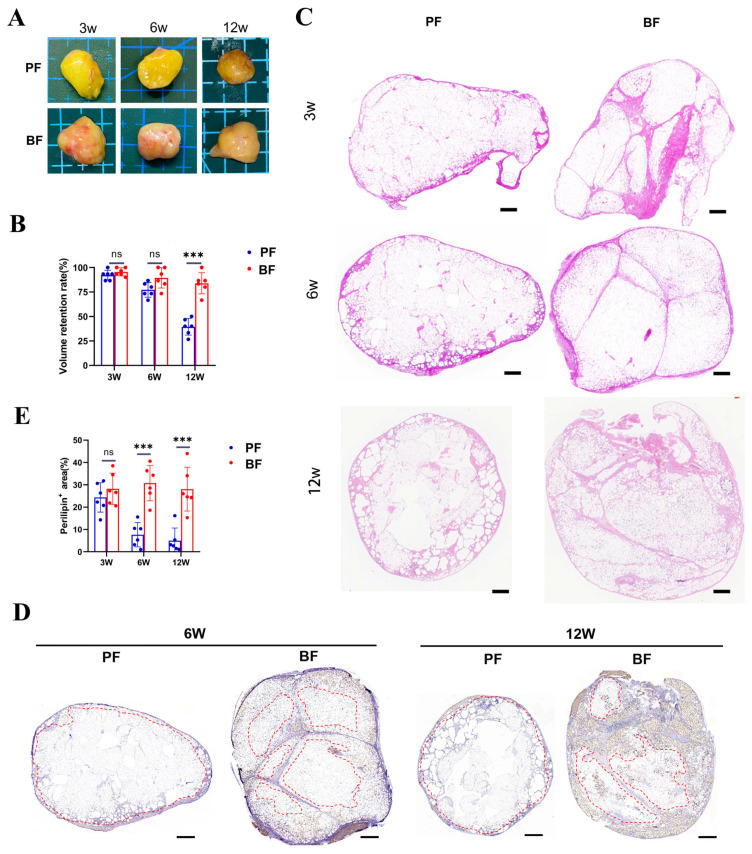
Transplantation of block fat (BF) and particle fat (PF) (n = 6, unpaired *t*-test). (**A**) Gross appearance of the transplanted grafts (scale bar: 0.5 cm). (**B**) Volume retention rate of transplanted grafts. (**C**) Hematoxylin and eosin (H&E) staining of the largest cross-sectional area of the PF (left column) and BF (right column) group. (**D**) Perilipin immunohistochemical staining of the largest cross-sectional area of PF (left column) and BF (right column) grafts. The red line encircles the perilipin-negative regions, which correspond to areas of dead adipocytes. (**E**) Percentage of perilipin-positive area. ***, *p* < 0.001; ns, not significant. Scale bars: 500 μm (**C**); 100 μm (**D**).

The volume of BF grafts did not show a significant decrease 12 weeks post-grafting (Figure 1A,B). Although there was no significant difference in volume retention between BF and PF at 3 and 6 weeks (Figure 1A,B), BF exhibited significantly higher retention at 12 weeks (Figure 1A,B). In HE staining, PF lacked a reticular LFC structure, and adipocytes formed more oil cysts, resulting in a more fragmented tissue appearance; in contrast, BF contained intact LFC that compartmentalized the adipocytes, with no significant formation of oil cysts (Figure 1C). Graft survival was assessed based on structural integrity, necrosis, vacuolization, fibrosis, and inflammatory response [17] in HE staining shown in Figure 1C, and the BF group scored better than the PF group at both 6 and 12 weeks (Table 1).

At 6 and 12 weeks, in contrast to PF, where perilipin+ cells were primarily distributed only around the periphery, forming a shell-like survival pattern, BF grafts showed no significant changes in the adipose fibrous connective tissue (Figure 1D), and perilipin+ cells in BF grafts were distributed around the LFC, both in the center and periphery, forming a mesh-like survival pattern (Figure 1D). The percentage area of perilipin+ cells was significantly greater in BF at 6 and 12 weeks (Figure 1E). Given the observed differences in survival between adipose tissues from the ALFC and DLFC regions, we hypothesized that the inherent properties of adipocytes or the post-transplant microenvironment might vary between these two areas.

### 3.2. Characteristics of Adipocytes and ADSCs Show No Significant Differences

#### 3.2.1. ALFC-Adipocyte and DLFC-Adipocyte

**Figure 2:**
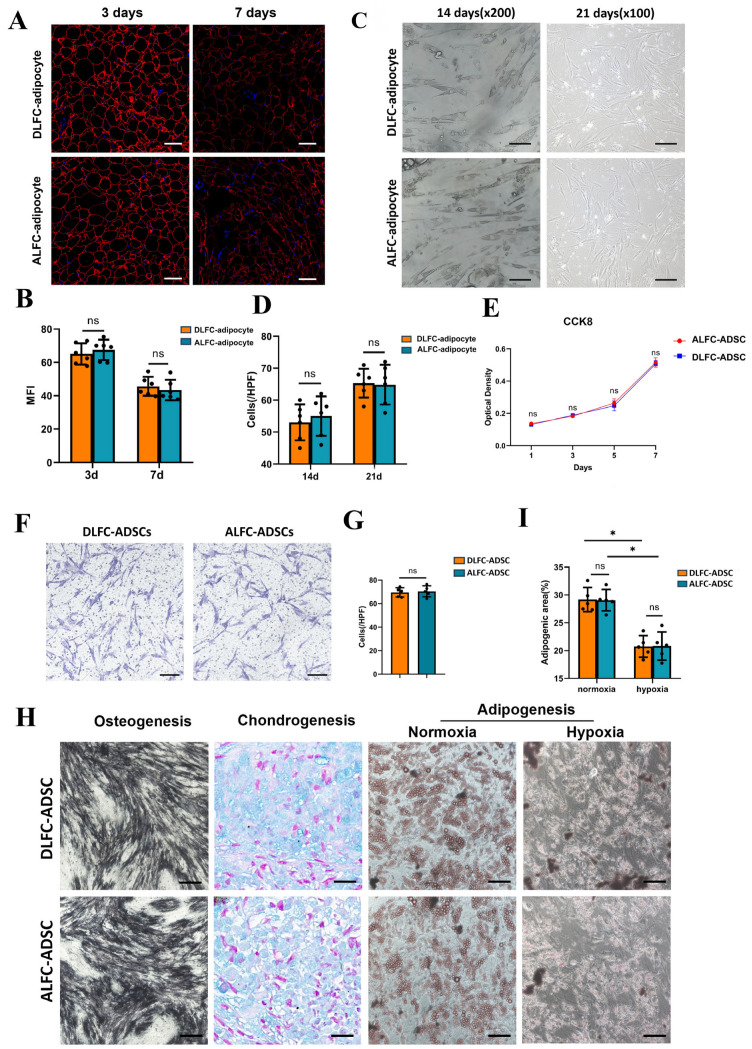
Characterization of adipocytes and adipose-derived stem cells (ADSCs) in areas adjacent (ALFC) and distant (DLFC) to large fibrous connective tissue (LFC) (n = 5, unpaired *t*-test). (**A**,**B**) Perilipin immunofluorescence of adipose tissue after 3 days and 7 days of incubation in serum-free medium under 1% volume fraction oxygen. (**C**,**D**) Dedifferentiation of mature adipocytes after 14 days and 21 days of ceiling culture. (**E**) Proliferative capacity of ADSCs by CCK-8 test. (**F**,**G**) Cell migration ability of ADSCs within 24 h. (**H**) Chondrogenic, osteogenic, and adipogenic differentiation potential of ADSCs under normoxic (21% O_2_) and hypoxic (1% O_2_) conditions. (**I**) Comparison of adipogenic area stained with Oil Red O. *, *p* < 0.05. ns: Not significant. Scale bar: 100 μm (**A**); 10 μm ((**C**) (200×)); 20 μm ((**C**) (100×)); 20 μm (**F**); 20 μm (**H**).

We examined the properties of adipocytes from both regions. Initially, we assessed their tolerance to hypoxia. After 3 and 7 days of hypoxic treatment, perilipin staining revealed no significant difference between the two groups (Figure 2A,B). The hypoxic environment following fat transplantation can induce dedifferentiation of adipocytes, reducing oxygen consumption and enhancing hypoxia tolerance, which benefits graft survival [15]. Following 14 and 21 days of dedifferentiation induction, adipocytes in both groups exhibited a spindle-shaped, multi-vesicular appearance (Figure 2C), with no significant difference in cell density (*p* > 0.05) (Figure 2D), indicating similar dedifferentiation abilities.

#### 3.2.2. ALFC-ADSCs and DLFC-ADSCs

Next, we investigated the properties of ADSCs from both regions. There was no significant difference in proliferation capacity over 1–7 days (Figure 2E) or in migration ability (Figure 2F,G). The adipogenic, chondrogenic, and osteogenic differentiation capacities after induction also showed no significant differences (Figure 2H). Under hypoxic conditions, the maximum diameter of lipid droplets and the lipid droplet area were significantly smaller compared with normoxic conditions, but there was no significant difference between the two groups (Figure 2H,I).

### 3.3. ALFC-ECM Contains More Mitochondria and Redox-Related Proteins than DLFC-ECM

**Figure 3:**
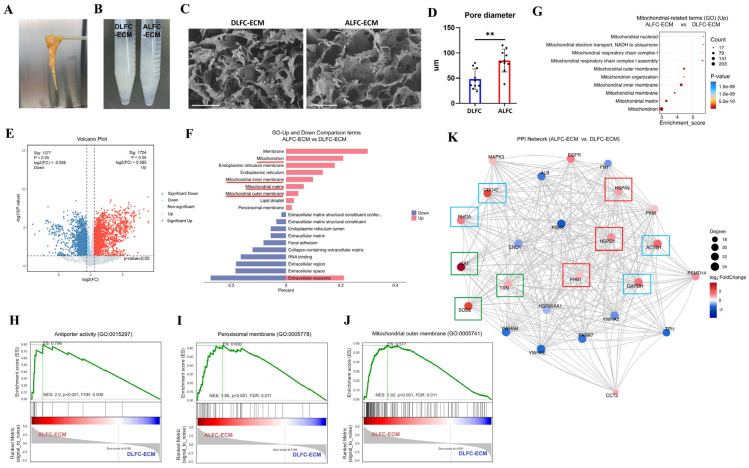
Proteomics analysis of extracellular matrix (ECM) components in adipose tissue adjacent (ALFC-ECM) and distant (DLFC-ECM) from large fibrous connective tissue (LFC) (n = 10, unpaired *t*-test). (**A**) Isolation of LFC and its surrounding adipose tissue from lipoaspirate. (**B**) Appearance of decellularized DLFC-ECM and ALFC-ECM. (**C**) SEM images. (**D**) Comparison of pore diameter. (**E**) Volcano plot showing differentially expressed ECM proteins. (**F**) Gene ontology (GO) analysis of the top 10 significantly upregulated and downregulated terms, with terms related to mitochondria underlined in red. (**G**) GO enrichment analysis displaying the top 10 most significantly differentially regulated terms related to mitochondria. (**H**–**J**) Gene set enrichment analysis (GSEA) of the three pathways with the highest fold change among those upregulated. (**K**) Protein–protein interaction (PPI) network, with mitochondria-related proteins highlighted in red boxes, redox-related proteins in green boxes, and cytoskeleton-related proteins in blue boxes. ** *p* < 0.01. Scale bar: 100 μm (**C**).

Given the lack of differences in cell intrinsic properties, we hypothesized that the disparities might reside within the microenvironment. Adipose tissue obtained through liposuction was processed to isolate the LFC along with the surrounding fat tissue as the ALFC group (Figure 3A), while the remaining fat tissue after LFC removal constituted the DLFC group. Both groups underwent decellularization to remove cellular and lipid components, yielding ECM that appeared macroscopically identical (Figure 3B). SEM revealed larger pores in the ALFC group (Figure 3C,D). Comparative proteomics of ECM components identified significant differences in protein composition, with 3101 proteins showing significant variations in abundance (Figure 3E).

Functional enrichment analysis indicated that the most significantly different proteins were associated with mitochondria (Figure 3F). The top 10 mitochondrial-related terms with the highest significance in GO analysis are shown in Figure 3G. GSEA analysis showed that the three most significantly increased terms in ALFC-ECM compared with DLFC-ECM were antiporter activity (GO:0015297), peroxisomal membrane (GO:0005778), and mitochondrial outer membrane (GO:0005741) (Figure 3H–J). Protein–protein interaction (PPI) network analysis demonstrated that the most closely interacting proteins significantly increased in the ALFC-ECM group was associated with mitochondria (red box), redox reactions (green box), and the cytoskeleton (blue box) (Figure 3K). Among the redox-related interacting proteins, catalase (CAT) (P04040), superoxide dismutase (SOD) (P04179), and thioredoxin (TXN) (P10599) were identified. CAT, with the largest fold change, is located in the matrix of peroxisomes [18] and functions to protect cells from the toxic effects of hydrogen peroxide [19]. SOD 2, located in the mitochondrial matrix [20], is responsible for scavenging toxic ROS [21]. These results suggest that ALFC-ECM contains more mitochondria than DLFC-ECM, indicating potentially more active metabolic processes in the LFC area.

### 3.4. Different Mitochondrial Distribution Between ALFC and DLFC Adipocytes After Grafting

**Figure 4:**
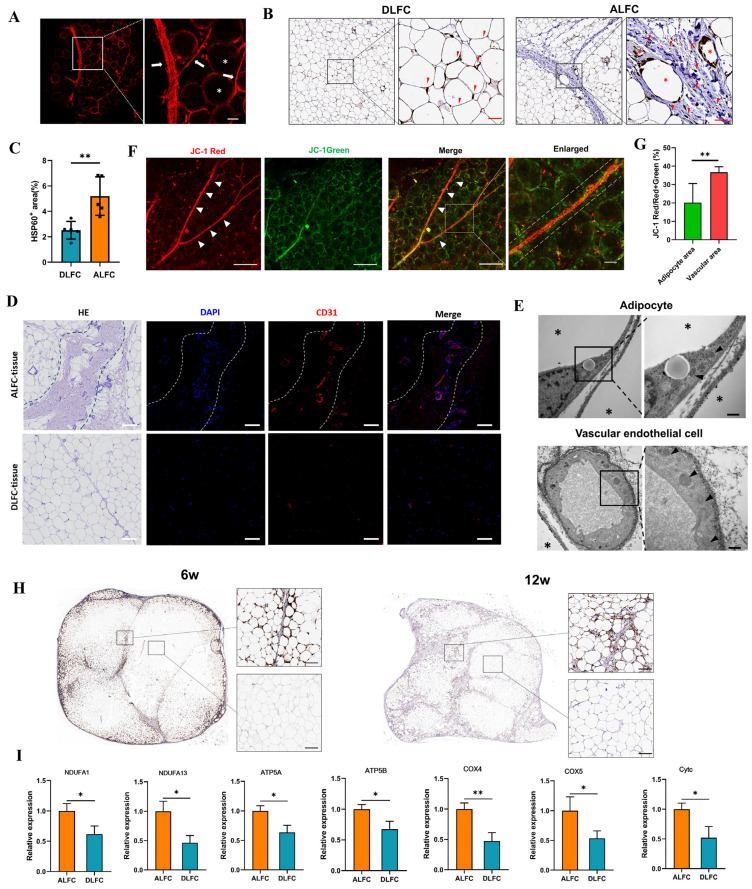
Mitochondrial distribution and function in adipose tissue before and after transplantation (n = 5, unpaired *t*-test). (**A**). MitoBright Red staining of adipose tissue. Arrows indicate regions of higher mitochondrial density with tubular distribution; asterisks indicate the lipid droplets in adipocytes, which do not contain mitochondria. (**B**) HSP60 immunohistochemical staining of adipose tissue adjacent (ALFC) and distant to large connective tissue (DLFC). Red triangles: mitochondria; asterisks: vessels in LFC. (**C**) Comparison of HSP60+ area between ALFC and DLFC (n = 5, unpaired *t*-test). (**D**) Hematoxylin and eosin (H&E) and CD31 staining of adipose tissue in ALFC and DLFC. Dashed lines outline the LFC region. (**E**) Transmission electron microscopy (TEM) of adipose tissue. Asterisks: lipid droplets; arrows: mitochondria. (**F**) JC-1 staining of adipose tissue. Triangles: mitochondria with high membrane potential in linear distribution. (**G**) Quantification of JC-1 fluorescence intensity between the vascular area and the adipocyte area (n = 5, unpaired *t*-test). (**H**) HSP60 immunohistochemical staining of block fat graft at 6 weeks and 12 weeks post-transplantation. (**I**) The mRNA expression levels of mitochondrial electron transport chain (*NDUFA1*, *NDUFA13*, *ATP5A*, *ATP5B*, *COX4*, *COX5*, *Cytc*) in ALFC and DLFC (n = 3, unpaired *t*-test). * *p* < 0.05, ** *p* < 0.01. Scale bars: 50 μm (**A**); 50 μm (**B**); 100 μm (**D**); 200 nm (**E**); 200 μm (**F**); 50 μm (F-Elarged); 100 μm (**H**).

In freshly obtained adipose tissue, mito-tracker immunofluorescence showed that the LFC contained a higher density of mitochondria (Figure 4A), which were arranged in a linear pattern. HSP60 staining showed positivity in the LFC and vessel walls within the ALFC, while primarily adipocyte nuclei were positive in the DLFC (Figure 4B). The proportion of positive areas in the ALFC and DLFC revealed that the density of mitochondria in the ALFC region was significantly higher than in the DLFC region (Figure 4C). CD31 staining revealed that the LFC contained blood vessels, whereas the non-LFC region had fewer vessels (Figure 4D). It is well established that white adipocytes are characterized by a low content of mitochondria [22]. TEM showed that, in adipocytes, mitochondria are primarily located in the cytoplasm surrounding the nucleus with a fragmented outer membrane, and that no mitochondria were observed within the lipid droplets; in endothelial cells, mitochondria are evenly distributed throughout the cytoplasm and have a more intact outer membrane (Figure 4E). Regarding MMP, JC-1 staining showed that mitochondria arranged in a linear pattern along the blood vessels in the connective tissue were red, indicating a higher MMP; in contrast, mitochondria arranged in a circular pattern within adipocytes in both ALFC and DLFC regions were predominantly green, indicating a lower MMP (Figure 4F). Quantification of JC-1 fluorescence intensity showed no statistically significant difference in the red/(red + green) ratio between the vascular area and the adipocyte area (Figure 4G). Six or twelve weeks after BF transplantation, the ALFC region showed significantly more HSP60-positive cells compared with the DLFC region (Figure 4H). We further evaluated the expression of genes related to the mitochondrial electron transport chain. The results show that, compared with ALFC, *NDUFA1*, *NDUFA13*, *ATP5A*, *ATP5B*, *COX4*, *COX5*, and *Cytc* were all significantly downregulated in DLFC (Figure 4I). These results indicate that under physiological conditions, the LFC contains a greater number and density of mitochondria, which are more functionally active, primarily located in the blood vessels of the LFC. The mitochondrial distribution in adipocytes was similar in ALFC and DLFC regions under physiological conditions but was significantly higher in the ALFC regions after grafting.

### 3.5. ALFC-ECM and DLFC-ECM Show No Significant Difference in Inducing Adipose Tissue Regeneration

**Figure 5:**
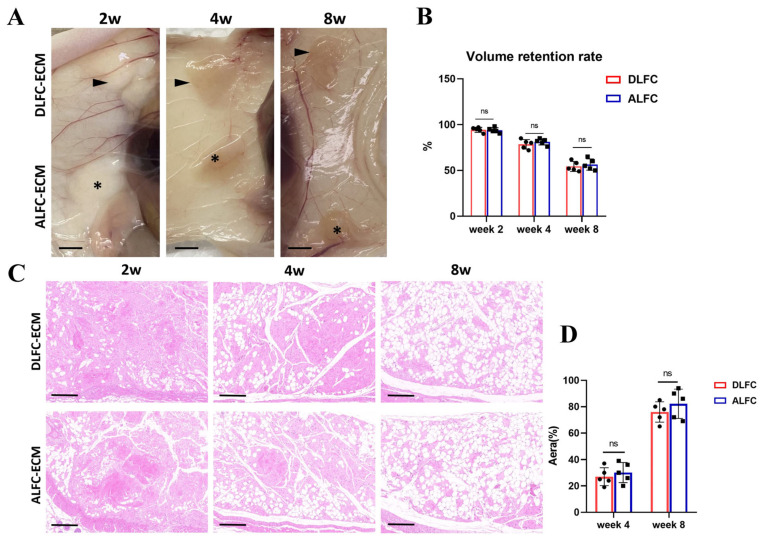
Comparison of the adipogenic capability of extracellular matrix (ECM) adjacent and distant from large fibrous connective tissue (LFC) (n = 5, unpaired two-tailed *t*-test). (**A**,**B**). Arrows: Gross photos of DLFC-ECM adipogenesis; Asterisks: Gross photos of ALFC-ECM adipogenesis. Gross appearance and volume retention rate of the scaffolds implanted in vivo at 2, 4, and 8 weeks. (**C**,**D**). Hematoxylin and eosin (H&E) staining and comparison of adipogenic area of the scaffolds implanted in vivo at 2, 4, and 8 weeks. ns: Not significant. Scale bars: 500 μm (**A**); 200 μm (**C**).

One mechanism of fat survival after transplantation is regeneration, involving the differentiation of ADSCs from the graft itself or host into mature adipocytes [17,22]. Various adipogenic factors in the ECM can induce adipogenesis [23]. We investigated whether ALFC-ECM had a stronger ability to induce adipose tissue regeneration compared with DLFC-ECM. There was no significant difference in the volume retention rate at 2 weeks, 4 weeks, and 8 weeks (Figure 5A,B), nor in the adipogenic area (Figure 5C,D). This suggests that the impact of the LFC in block fat on fat transplantation is not related to adipose tissue regeneration.

### 3.6. Mitochondrial Transplantation Can Promote Survival of Transplanted Fat by Reducing Oxidative Stress in the Early Stage

#### 3.6.1. Free Mitochondria Survive Only Short-Term After Transplantation

**Figure 6:**
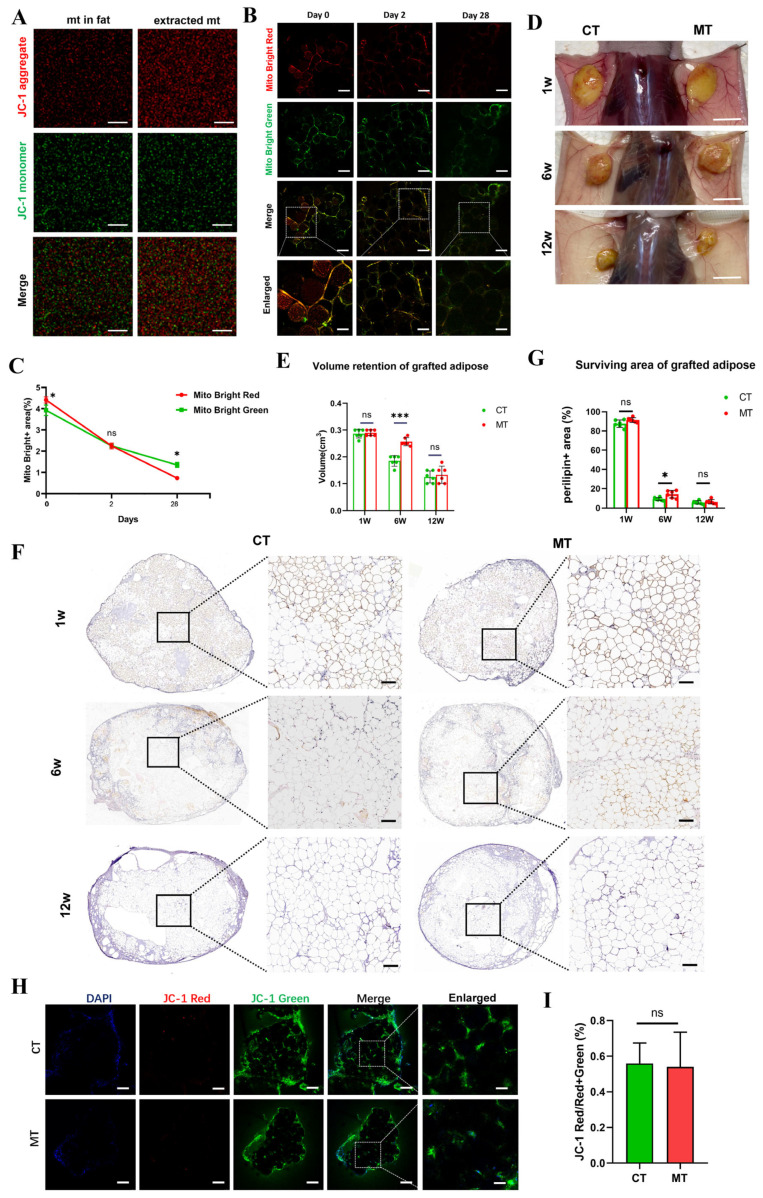
Impact of mitochondrial transplantation on the survival of fat grafts (n = 6, unpaired *t*-test). (**A**) Membrane potential of mitochondria isolated from adipose tissue and from adipose-derived stem cells. mt: mitochondria. (**B**,**C**) Survival of native adipose mitochondria and transplanted mitochondria in adipose grafts at day 0, 2, and 28 post-mitochondrial transplantation. Mito Bright Red: transplanted mitochondria; Mito Bright Green: native adipose mitochondria. (**D**,**E**) Survival of fat grafts in the mitochondrial transplantation (MT) group and the control (CT) group at 1 week, 6 weeks, and 12 weeks post-transplantation. (**F**,**G**) Perilipin immunohistochemical staining and percentage of perilipin-positive area in both groups. (**H**,**I**) JC-1 staining of MT and CT group at 4 weeks post-transplantation and quantification of JC-1 fluorescence intensity. * *p* < 0.05, *** *p* < 0.001, ns: Not significant. Scale bars: 10 μm (A); 100 μm (**B**); 50 μm ((**B**) enlarged); 1 cm (**D**); 200 μm (**F**); 100 μm (**H**); 30 μm ((**H**) enlarged).

Given the significant differences in mitochondrial density and function between ALFC and DLFC, we aimed to directly explore whether mitochondria could promote the survival of transplanted fat. Before transplantation, the MMP was detected for both mitochondria obtained from ADSCs and from adipose tissue (Figure 6A). On the day of grafting (day 0) and 2 days post-grafting, red-labeled transplanted mitochondria and green-labeled endogenous mitochondria were visible, and the latter exhibited higher fluorescence intensity (Figure 6B,C). At 28 days, under equivalent fluorescence quenching, the decrease in fluorescence of the mitochondria transferred group was significantly higher than that of the host’s mitochondria, but there was no significant difference in the degree of decrease between low and high concentrations of transplanted mitochondria (Figure 6B,C, Supplemental Figure S1A). This indicates that transplanted mitochondria only survive for a shorter period compared with in situ mitochondria, and this is independent of the concentration of transplanted mitochondria.

#### 3.6.2. Mitochondrial Transplantation Increases Early Volume Retention Rate

The volume retention rate of transplantation with a higher concentration of mitochondria significantly increased the volume of particulate fat up to 6 weeks (Figure 6D,E), while lower concentrations had no significant effect (Supplemental Figure S1B,C). Therefore, only the high concentration group was used for follow-up experiments. At 6 weeks post-grafting, perilipin+ adipocytes were present both in peripheral and internal areas in the mitochondrial transplant (MT) group, whereas perilipin+ adipocytes in the control (CT) group were only present at the edges (Figure 6F). The total area of perilipin+ adipocytes was significantly greater in the MT group at 6 weeks (Figure 6G). However, at 12 weeks post-grafting, there was no difference in volume retention or adipocyte survival between the MT and CT group; the adipocytes in both groups survived at the periphery of the graft but died in the central region. (Figure 6D–G). JC-1 staining of the grafts at 4 weeks showed that the MMP in both groups exhibited relatively low green fluorescence, and that there was no statistically significant difference in the ratio of red to red + green between the groups (Figure 6H,I).

#### 3.6.3. Transplanted Mitochondria Reduce Inflammation by Clearing ROS

**Figure 7:**
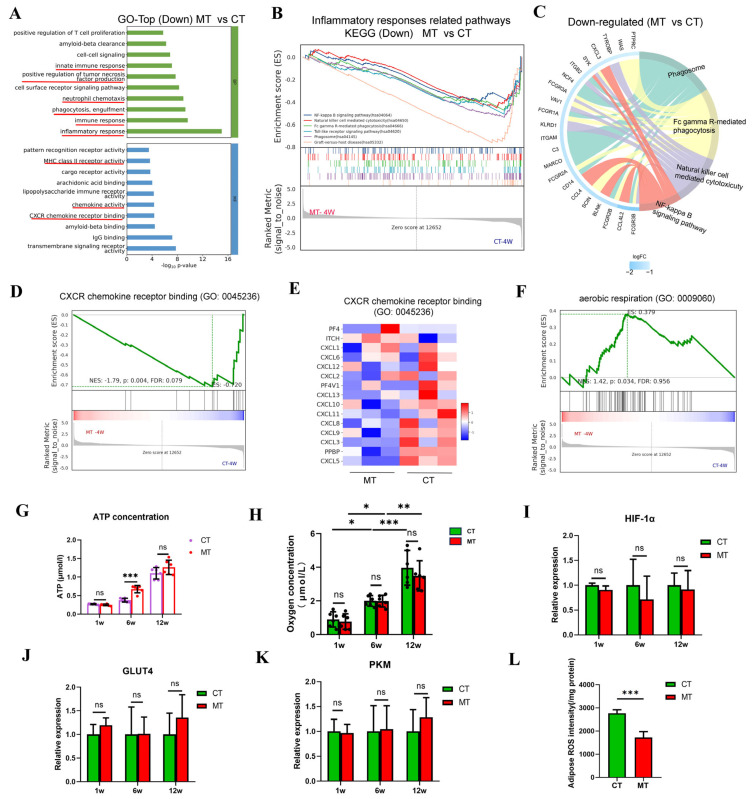
Mitochondrial transplantation reduces oxidative stress and immune response and improves short-term volume retention in fat grafts. (**A**) Gene ontology (GO) enrichment analysis showing the top 10 significantly downregulated terms in biological processes and molecular functions. Terms related to immune inflammation are highlighted in red. (**B**) Gene set enrichment analysis (GSEA) of significantly downregulated inflammatory response pathways. (**C**) Kyoto Encyclopedia of Genes and Genomes (KEGG) analysis showing significantly downregulated inflammatory response pathways represented in a chord diagram. (**D**,**E**) GSEA showing significant downregulation of the CXCR chemokine receptor binding pathway. (**F**) GSEA showing the aerobic respiration term. (**G**) ATP concentration in grafts from the CT and the MT groups post-transplantation (n = 6, unpaired *t*-test). (**H**) Oxygen concentration in the center of grafts (n = 6, unpaired *t*-test). (**I**–**K**) Expression levels of HIF-1α, GLUT4, and PKM genes in the CT and MT groups (n = 3, unpaired *t*-test). (**L**) Adipose ROS concentration at 4 weeks post-transplantation in the CT and MT groups (DHE probe fluorescence intensity to protein concentration, n = 5, Student’s *t* test). * *p* < 0.05, ** *p* < 0.01, *** *p* < 0.001, ns: No significance.

To investigate the mechanism by which transplanted mitochondria promote the survival of transplanted fat, transcriptome analysis was performed on the MT and CT groups 4 weeks post-transplantation. GO enrichment and GSEA analysis showed that the most significantly downregulated terms in the MT group were related to immune-inflammatory responses (Figure 7A,B). KEGG analysis revealed significant downregulation of pathways, including phagosome (hsa04145), natural killer cell-mediated cytotoxicity (hsa04650), Fc gamma R-mediated phagocytosis (hsa04666), and the NF-kappa B signaling pathway (hsa04064) (Figure 7C). CXCR chemokine receptor binding (GO: 0045236) was also significantly downregulated (Figure 7D,E). These findings indicate that the primary effect of mitochondrial transplantation is a reduction in inflammatory responses.

We hypothesized that the increased mitochondrial content could enhance oxygen uptake and mitigate hypoxic damage, thereby helping maintain cellular homeostasis and reduce inflammation caused by cell necrosis. However, GO analysis of response to hyperoxia (GO:0055093) did not reveal significant differences (Supplemental Figure S1D), and while aerobic respiration (GO:0009060) showed significant differences, the FDR was over 0.25 (Figure 7F). Measurement of ATP showed an increase in the MT group at 6 weeks (Figure 7G). Measurements of oxygen concentration in the grafts showed a gradual increase from 1 week to 12 weeks, but the oxygen levels remained below the subcutaneous oxygen concentration of 10 μmol/L in nude mice, and there were no significant differences between the groups (Figure 7H, Supplemental Figure S1E). PCR analysis of genes in hypoxia response, glucose uptake, and glycolysis-related genes did not reveal significant differences (Figure 7I–K). Hypoxic conditions can lead to oxidative stress and the production of ROS [24], which can trigger inflammatory responses [25]. Mitochondria are the primary site for the clearance of ROS [26]. The relative intensity of ROS (DHE probe fluorescence intensity to protein concentration) in the grafts 4 weeks after grafting was significantly lower in the MT group than in the CT group (Figure 7L). These results suggest that transplanted mitochondria do not significantly affect cellular energy metabolism but rather can clear ROS produced and accumulated under hypoxic conditions, thereby reducing cellular stress damage and inflammation. Furthermore, this effect does not depend on oxygen concentration.

### 3.7. Long-Term Survival of Mitochondria in the LFC Promotes Long-Term Fat Survival

The establishment and stabilization of blood supply in fat grafts typically take about three months, making the long-term effects of mitochondria necessary. In our block fat samples, we observed that the advantages in volume retention persisted at three months, with surviving adipocytes growing around the LFC. From Section 3.4, we found that the LFC region contains a large number of mitochondria, leading us to hypothesize that the LFC can act as a carrier for these mitochondria, allowing them to survive long-term post-transplantation and help reduce inflammatory responses.

**Figure 8:**
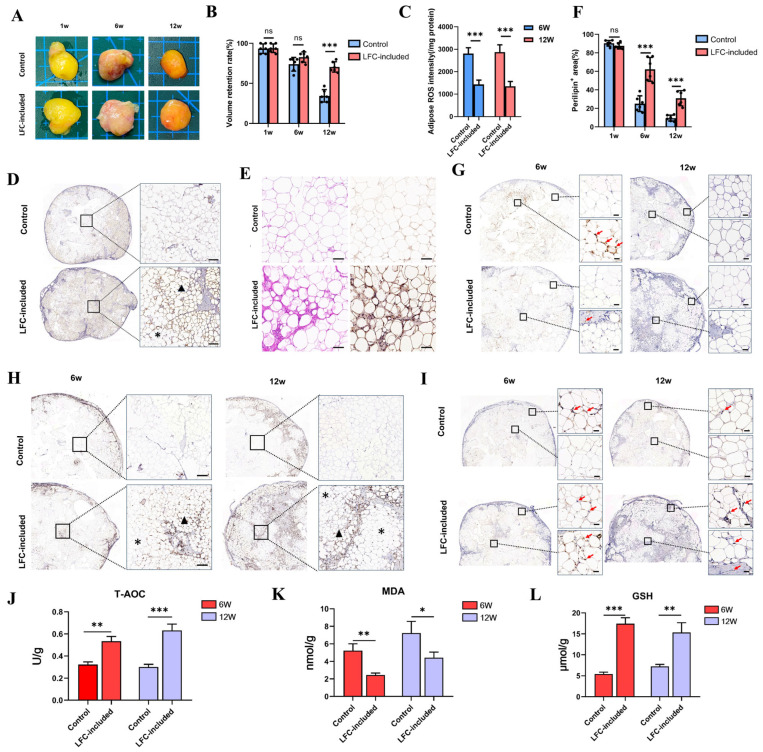
Large fibrous connective tissue (LFC) enhances long-term survival of fat grafts. Gross appearance of the fat graft in the Control and LFC-included groups (**A**) and volume retention rate (**B**) (scale bar: 0.5 cm, n = 6, unpaired *t*-test). (**C**) Intensity of reactive oxygen species (ROS) at 6 and 12 weeks post-transplantation (n = 5, unpaired *t*-test). (**D**) Perilipin immunohistochemistry at 6 weeks showing differences in adipocyte survival near LFC (triangles) and distant from LFC (asterisks). (**E**) HE & perilipin immunofluorescence at 12 weeks. (**F**) Percentage of perilipin-positive cell area at 1 week, 6 weeks, and 12 weeks post-transplantation (n = 6, unpaired *t*-test). (**G**) Ly6G immunohistochemistry at 6 weeks and 12 weeks post-transplantation. Red arrows indicate Ly6G-positive cells. (**H**) HSP60 immunohistochemistry at 6 and 12 weeks post-transplantation. Triangles: adipocyte adjacent to LFC; asterisks: adipocyte distant from LFC. (**I**) SOD1 immunohistochemistry at 6 weeks and 12 weeks post-transplantation. Red arrows indicate SOD1-positive cells. (**J**–**L**) Relative activities of T-AOC and relative concentrations of GSH and MDA in adipose grafts (n = 3, unpaired *t*-test). * *p* < 0.05, ** *p* < 0.01, *** *p* < 0.001, ns: Not significant. Scale bars: 200 μm (**D**); 100 μm (**E**); 50 μm (**G**); 100 μm (**H**); 50 μm (**I**).

When LFC was mixed with particulate fat, it significantly improved the early and late volume retention rates of the grafts (Figure 8A,B). The intensity of ROS at 6 and 12 weeks was significantly lower in the LFC-included group compared with the control (Figure 8C). The area occupied by perilipin+ cells in the LFC-included group was significantly larger at both 6 weeks (Figure 8D) and 12 weeks (Figure 8E,F), with cells present in the center and surrounding the LFC; here, the structure of the adipocytes is clearer, with a visible abundance of small adipocytes. In contrast, the control group lacked viable perilipin+ cells in the center, and, at 6 weeks, necrotic and disintegrated adipocytes were visible in the center and small adipocytes were not observed (Figure 8D). Neutrophil staining showed that, at 6 weeks, the central region of the control group had significantly more neutrophils than the peripheral region, while the LFC-included group had fewer neutrophils in both regions (Figure 8G). By 12 weeks, both groups had fewer neutrophils (Figure 8G).

Next, we examined the survival and function of mitochondria in the grafts. In the control group, no mitochondria were detected in the central region at 6 weeks and 12 weeks (Figure 8H). In contrast, mitochondria in the LFC were detectable at 6 weeks and 12 weeks post-transplantation, with a significantly higher content of mitochondria in the ALFC region’s adipocytes (triangles) compared with the DLFC region (stars) (Figure 8H). Comparison of the redox state of the grafts showed that LFC significantly increased the T-AOC of the graft, enhanced GSH expression, and reduced MDA levels (Figure 8I–K). The expression of SOD-1, a key enzyme involved in ROS clearance, was significantly higher in the LFC-included group compared with the control group at both 6 weeks and 12 weeks (Figure 8L). In the control group, SOD-1 expression was only present in the periphery, whereas in the LFC-included group, the positive areas were observed in both the periphery and the center, particularly in ALFC adipocytes (Figure 8L). These findings demonstrate that mitochondria attached to the LFC can survive long-term and can clear ROS through enzymes like SOD-1, thereby improving the long-term volume retention of transplanted fat.

## 4. Discussion

Fat grafting is a common procedure in clinical practice, yet achieving long-term volume retention remains a significant challenge. Previous studies have attempted to identify the optimal methods for harvesting fat and injection techniques, but the best approach remains controversial [26]. Other research has involved adding ADSCs [27] or platelet-rich plasma [18,19] to fat grafts in order to improve volume retention, but these methods have not shown significant effects. Our research introduces a novel paradigm for fat survival that centers around LFC (large connective tissue) that is mesh like, deviating from the traditional shell-like survival pattern and breaking through the limitations imposed by the ischemic and hypoxic gradients determined by tissue fluid diffusion distances. The mechanism involves LFC equipped with high-quality mitochondria that can survive under hypoxic conditions following transplantation, reducing oxidative stress damage through enzymes such as SOD1. This study highlights the pivotal role of LFC in fat transplantation, overcoming the limitations imposed by the ischemic and hypoxic gradient determined by distance and interstitial fluid penetration, providing a new perspective on enhancing long-term stability and clinical feasibility. Moreover, it is important to acknowledge the potential variability in LFC effectiveness, which can differ between individuals and across various clinical conditions.

Ischemia and hypoxia after transplantation are inevitable. The MMP is formed by the proton gradient across the inner membrane, which is essential for ATP synthesis [27]. MMP maintains the function of the electron transport chain (ETC), enabling the transfer of electrons from NADH and FADH2 to oxygen, ultimately producing water and driving ATP synthase to generate ATP. Within the mitochondria, the ETC is also a major source of reactive oxygen species (ROS). Changes in MMP may indicate that the cell is under stress or experiencing a potential pathological condition [28]. Hypoxia leads to mitochondrial dysfunction and ATP depletion, triggering a cascade of events including excessive production of ROS and calcium overload [29]. Damaged mitochondria are the primary source of ROS production. When the balance between ROS generation and their removal by cellular antioxidant systems is disrupted, oxidative stress occurs [30], damaging cellular components such as lipids, proteins, mitochondria, and DNA [31], leading to cell injury and apoptosis [32]. Timely clearance of accumulated ROS during the ischemic and hypoxic period is beneficial for promoting the survival of fat grafts [33,34]. The primary sites for ROS clearance are the mitochondria and peroxisomes [35,36], primarily through enzymes such as superoxide dismutase [37]. In our results, the mitochondrial density in the ALFC region was significantly higher than in the DLFC region, and the PPI analysis showed that the most closely interacting proteins among those significantly increased were redox-related proteins. This indicates that the mitochondria in the ALFC region can more effectively clear ROS. Numerous data show that mitochondria can be actively released by cells and transferred between cells in both healthy and pathological conditions [38,39], such as to cells with mitochondrial dysfunction [40]. Damaged cells can also take up exogenous functional mitochondria from donor cells and integrate them into their endogenous mitochondrial network to improve biological processes [41,42]. In our results, the MMP in LFC was significantly higher than in adipocytes before transplantation, and the RNA expression of mitochondrial respiratory chain enzymes was also higher, suggesting stronger mitochondrial function. After transplantation, more viable adipocytes were observed around the LFC, and co-transplantation of LFC with adipose tissue resulted in higher SOD1 levels and lower ROS and inflammation. This means the mitochondria in LFC can better tolerate the hypoxic environment after transplantation and can be taken up by surrounding adipocytes experiencing mitochondrial dysfunction due to oxidative stress, improving these cells’ ability to clear ROS. Conversely, DLFC adipocytes, due to their distance, cannot take up mitochondria from LFCs and thus die due to higher oxidative stress damage and inflammation. The mitochondrial density of adipocytes in the ALFC region after transplantation was significantly higher than that before transplantation, suggesting that mitochondrial transfer may have occurred. This explains why surviving adipocytes are distributed around the LFC. Despite the promising advantages of LFC observed in our study, the mitigation of ischemia and hypoxia through LFC requires validation in larger clinical trials to confirm these potential benefits.

To ensure that all regions of the graft benefit from high-quality mitochondria, we extracted mitochondria from ADSCs and mixed them evenly with adipocytes through co-culture in vitro, achieving mitochondrial transplantation. The concept of mitochondrial transplantation was first proposed by Clark and Shay in 1982 [43]. In 2009, McCully and colleagues reported the first in vivo study of mitochondrial transplantation [44]. Mitochondrial transplantation has shown promising therapeutic effects in various types of mitochondrial dysfunction diseases, including myocardial infarction, ischemic stroke, Alzheimer’s disease, acute spinal cord injury, acute lung disease, and even cancer [45]. Its main effects are the reduction of oxidative stress [46] through overexpression of antioxidant enzymes [47]. Currently, there are no reports of mitochondrial transplantation in the field of fat grafting.

We utilized ADSCs-derived mitochondria and transplanted them into adipose grafts, observing a significantly enhanced volume retention rate and reduced inflammation and ROS levels at six weeks. We confirmed that these mitochondria from stem cells had a higher membrane potential than those from adipocytes in lipoaspirates, which may be a prerequisite for their uptake and function post-implantation. Stem cells are favored as a mitochondrial source due to their abundant mitochondria, superior self-renewal capacity, and low levels of oxidative damage [48,49], further highlighting their therapeutic potential [50]. However, studies have reported that donor mtDNA is undetectable by day seven post-transplantation, indicating the limited survival of transplanted mitochondria [51]. Further research found that mtDNA-depleted mitochondria still significantly enhance the angiogenic capacity of vascular endothelial cells, suggesting that even the “ghosts” of mitochondria can function, though the mechanism remains unknown [50]. We obtained similar results, implying that mitochondrial transplantation can only survive in the early stage. As angiogenesis increases, oxidative stress damage to adipocytes is alleviated [51]. However, our measurements of oxygen concentration within the adipose grafts showed hypoxia persisting for up to three months, suggesting that the short-term effects of mitochondria are insufficient to sustain the long-term survival of fat transplants. Moreover, isolated mitochondria maintain activity and coupling for approximately one hour on ice, after which storage significantly affects transplant efficacy [52]. These factors limit the clinical application of free mitochondrial transplantation.

Our results show that, when block fat transplantation was performed and mitochondria was retained within the LFC, the volume retention rate was improved for up to three months. Conventionally, non-adipocyte components, such as fascia and collagen fibers, are considered detrimental to fat survival and are typically removed. However, LFC not only provides structural support but also contains a vascular network that delivers nutrients and oxygen to cells. Studies have shown that ADSCs reside in the fascia and migrate into the adipose tissue as they mature [53]. Removing the fascia during the preparation of nanofat leads to a loss of approximately 40% of the stromal vascular fraction (SVF) [54]. When fascial tissue is mixed with a decellularized adipose matrix and transplanted, it significantly enhances the adipogenic area and vascularization of the decellularized matrix scaffold while reducing inflammation [55]. These studies, together with our findings, suggest that the role of LFC in fat transplantation and adipose regeneration should be reconsidered.

We found that LFC is a rich reservoir of mitochondria. Various cell types within the fascia, including fibroblasts, immune cells, and undifferentiated mesenchymal cells [56], can provide high-quality mitochondria. To ensure adequate contact between the LFC and fat particles without altering the texture of the fat graft, we minced the LFC into a paste. Compared with isolated mitochondrial transplantation, minced LFC transplantation resulted in surviving mitochondria within the LFC at 12 weeks, whereas the mitochondria in the DLFC region had already died. This might be because the vascular endothelial cells and ADSCs within the LFC are more resistant to ischemia and hypoxia than adipocytes [56]. Vascular endothelial cells within the LFC can secrete hypoxia-inducible factors, vascular endothelial growth factor (VEGF), and other factors that promote hypoxia tolerance, enhancing the hypoxia resistance of surrounding tissues [57]. Furthermore, mitochondria exposed to the extracellular environment are prone to death due to high calcium levels and pH changes [58]. Dead mitochondria act like ‘bombs’, releasing potassium ions that can affect the membrane potential and survival of surrounding cells [59]. The presence of LFC can effectively protect the mitochondria and enable them to function properly. This makes LFC an ideal carrier for mitochondrial transplantation, creating a suitable microenvironment and conditions for the transfer of mitochondria to adjacent adipocytes.

There are multiple methods for mitochondrial transplantation. The most recognized molecular mechanisms for intercellular mitochondrial transfer include tunneling nanotubes (TNTs) [60] and extracellular vesicles (EVs) [61], as well as co-culture [62], microinjection [63], and photothermal nanoblade delivery [64]. Compared with these methods, using autologous LFC as a carrier for mitochondrial transplantation offers several advantages. Firstly, it is easier to obtain and manipulate. Unlike extracting and culturing stem cells to isolate mitochondria, LFC can be obtained simultaneously with lipoaspirate and used immediately after simple processing, allowing for completion within a single surgical procedure without significantly increasing operation time. We recommend using a large-bore needle for liposuction to yield more LFC while reducing damage to fat particles [65]. Secondly, and most importantly, it is safer. On one hand, autologous LFC transplantation avoids the risk of rejection associated with allogeneic mitochondrial transplantation [66]. Mitochondria are highly immunogenic organelles [67]. In animal models, autologous mitochondrial transplantation has not significantly increased immune marker levels [68]. On the other hand, cultured stem cell-derived mitochondria carry additional risks such as bacterial contamination, fetal bovine serum antigens [69], and viral contamination [69].

Certainly, this study still has several issues that require further investigation. Firstly, the optimal ratio of LFC to adipocytes for mixing was not extensively investigated in this study and represents a direction for future research. Based on the results of free mitochondrial transplantation, we believe that a higher ratio of LFC would be more conducive to survival. However, the amount of LFC that can be obtained in a single liposuction procedure is limited in clinical practice. Secondly, it is unclear whether LFC might affect the hardness of the fat graft. Adipocyte necrosis-induced fibrosis can lead to nodule formation in the graft [70]. While our animal experimental results support the idea that LFC reduces graft inflammation and fibrosis, LFC itself is a fibrous component, and whether it affects the hardness of the graft requires further investigation. Thirdly, the process of cutting LFC into pieces and mixing it with adipose tissue may increase the risk of surgical contamination. Fourth, this study primarily used tissue from middle-aged and young female patients with specific BMIs, limiting its generalizability. Future studies should include subjects of different ages, BMIs, and genders to broaden the applicability of the findings.

## 5. Conclusions

This study introduces the novel concept of non-centripetal survival around large fibrous connective tissue (LFC) in fat grafting. We demonstrated that LFC can serve as an ideal carrier for a high density of functional mitochondria, which can survive under hypoxic conditions post-transplantation and reduce oxidative stress damage. By mixing LFC with particulate fat, we observed a significant improvement in the long-term volume retention and survival of adipocytes, as well as a reduction in inflammation and ROS levels. These findings suggest that LFC plays a crucial role in supporting adipocyte survival and offers a new mechanism for overcoming the limitations imposed by ischemic and hypoxic gradients in fat grafting. Incorporating LFC into fat grafting protocols may lead to improved outcomes in clinical applications.

## Figures and Tables

**Table 1:** Histologic scoring of particle fat graft and block fat graft.

	Particle Fat	Block Fat
Structural integrity		
3 weeks	4.1 ± 0.61	4.5 ± 0.49 *
6 weeks	3.30 ± 0.45	4.65 ± 0.47 ***
12 weeks	1.25 ± 0.42	4.30 ± 0.45 ***
Necrotic area		
3 weeks	1.1 ± 0.53	1.05 ± 0.49
6 weeks	4.10 ± 0.53	1.35 ± 0.47 ***
12 weeks	4.95 ± 0.21	2.75 ± 0.87 ***
Vacuoles		
3 weeks	0.6 ± 0.72	0.3 ± 0.45
6 weeks	2.40 ± 0.48	0.40 ± 0.48 ***
12 weeks	3.9 ± 0.53	2.45 ± 0.84 ***
Fibrosis		
3 weeks	0.9 ± 0.61	0.6 ± 0.48
6 weeks	2.15 ± 0.35	0.70 ± 0.44 ***
12 weeks	3.85 ± 0.35	2.45 ± 0.84 ***
Inflammation		
3 weeks	0.85 ± 0.64	0.4 ± 0.48 *
6 weeks	2.55 ± 0.49	0.5 ± 0.49 ***
12 weeks	2.6 ± 0.65	2.1 ± 0.61 *

The values in the table represent the mean ± SD. * Indicates *p* < 0.05 in histological scores compared with particle fat. *** Indicates *p* < 0.001 in histological scores compared with particle fat.

## Data Availability

Data are available and shared in iProx public database (Integrative Proteome Resource) (access link: https://ngdc.cncb.ac.cn/databasecommons/database/id/6181). The specific dataset identifier is OMIX 007122.

## Supplementary Materials

The following supporting information can be downloaded at: https://www.mdpi.com/article/10.3390/antiox14030270/s1, Figure S1: Fat grafting assisted by low and high concentrations of transplanted mitochondria. (**A**): Comparison of Retention Between Transplanted Mitochondria and Endogenous Mitochondria. (**B**): Macroscopic Appearance of the Fat Graft. (**C**): Volume retention rate between low and high concentrations of transplanted mitochondria; (**D**): GSEA analysis of response to hyperoxia (GO:0055093). E: Graft Oxygen Concentration Measurement. Table S1: Primer Sequences.
