# Supplementary material for: Large Fibrous Connective Tissue Reduces Oxidative Stress to Form a Living Cell Scaffold in Adipose Grafts
Source: Antioxidants (Basel). 2025 Feb 26;14(3):270. doi: 10.3390/antiox14030270 (PMC11939587; doi:10.3390/antiox14030270)
Supplement: Supplementary file 1 [file antioxidants-14-00270-s001.zip › Supplemental table.pdf]

Supplemental Table S1. Primer Sequences

Num	Gene Symbol	Forward primer(5->3)	Reverse primer(5->3)	mRNA accession number
1	HIF-1α	ATCAGAAGATACAAGTAGCCTC	TATGATTGTGTCTCCAGCGG	NM_001530.4
2	GLUT4	AGAGAAGAAACCAAAGGTCG	ATCCTCAAGTACCTCCACAATA	NM_001328496.2
3	PKM	GTGTGACGAGAACATCCTG	AATAAGCCCATCATCCACGTA	NM_002609.3
4	NDUFA1	GCTTCCGGGGAAGGAATCAA	CCGGGGAGAATTTCGAACCA	NM_001142566.2
5	NDUFA13	ATGAAGGATGTGCCGGACTG	CCATAGGTGGCGCTGAGAAT	NM_018996.4
6	ATP5A	ACGCCATTGATGGAAAGGG	TGGTTCCCGCACAGAGATTC	NM_001697.5
7	ATP5B	CATGTTGGGCTTTGTGGGTC	ATAGTCTCTGGCAGGCTGGA	NM_001700.3
8	COX4	CCAAGTGGGACTACGACAA	CCTGCTCGTTTATTAGCACTGG	NM_001867.4
9	COX5	ATCTGGAGGTGGTGTTCCTA	GTTGGTGATGGAGGGGACTAAA	NM_004374.4
10	Cytc	CTGGGGAGAGGAGACACTG	AGGCGGTGGCCAACTTTTAC	NM_001833.4
