# Supplementary figures and images for: Large Fibrous Connective Tissue Reduces Oxidative Stress to Form a Living Cell Scaffold in Adipose Grafts
Source: Antioxidants (Basel). 2025 Feb 26;14(3):270. doi: 10.3390/antiox14030270 (PMC11939587; doi:10.3390/antiox14030270)

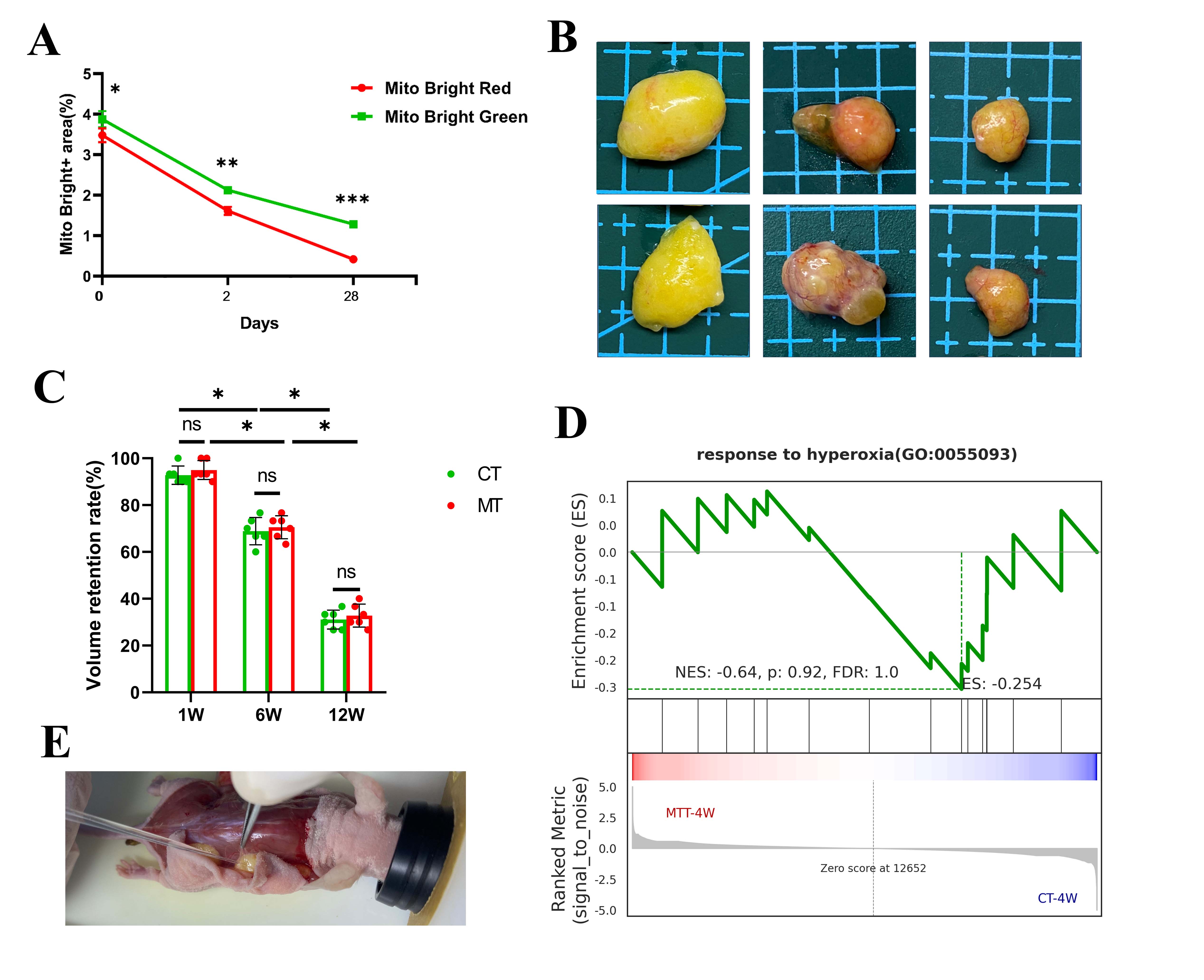

Supplement: Supplementary file 1 [file antioxidants-14-00270-s001.zip › Supplemental figure S1.jpg]
